# Genome region aware CADD thresholds for noncoding variant prioritization

**DOI:** 10.1093/nargab/lqaf157

**Published:** 2025-11-19

**Authors:** Jude-Félix Tenywa, Jean-Baptiste Lamouche, Sarah Baer, Samuel Nicaise, Antony Le Béchec, Amélie Piton, Jean Muller

**Affiliations:** Unité Fonctionnelle de Bioinformatique Médicale appliquée au diagnostic (UF7363), Hôpitaux Universitaires de Strasbourg, 67000 Strasbourg,France; Institut de Génétique et de Biologie Moléculaire et Cellulaire, INSERM U964, CNRS UMR7104, Université de Strasbourg, 67400 Illkirch,France; Unité Fonctionnelle de Bioinformatique Médicale appliquée au diagnostic (UF7363), Hôpitaux Universitaires de Strasbourg, 67000 Strasbourg,France; Institut de Génétique et de Biologie Moléculaire et Cellulaire, INSERM U964, CNRS UMR7104, Université de Strasbourg, 67400 Illkirch,France; Institut de Génétique et de Biologie Moléculaire et Cellulaire, INSERM U964, CNRS UMR7104, Université de Strasbourg, 67400 Illkirch,France; Unité Fonctionnelle de Bioinformatique Médicale appliquée au diagnostic (UF7363), Hôpitaux Universitaires de Strasbourg, 67000 Strasbourg,France; Unité Fonctionnelle de Bioinformatique Médicale appliquée au diagnostic (UF7363), Hôpitaux Universitaires de Strasbourg, 67000 Strasbourg,France; Institut de Génétique et de Biologie Moléculaire et Cellulaire, INSERM U964, CNRS UMR7104, Université de Strasbourg, 67400 Illkirch,France; Laboratoires de Diagnostic Génétique, IGMA, Hôpitaux Universitaires de Strasbourg, 67000 Strasbourg,France; Unité Fonctionnelle de Bioinformatique Médicale appliquée au diagnostic (UF7363), Hôpitaux Universitaires de Strasbourg, 67000 Strasbourg,France; Laboratoires de Diagnostic Génétique, IGMA, Hôpitaux Universitaires de Strasbourg, 67000 Strasbourg,France; Laboratoire de Génétique Médicale, UMR 1112, INSERM, IGMA, Université de Strasbourg, 67000 Strasbourg,France

## Abstract

The democratization of genome sequencing for genetic diseases is leading to the identification of a large amount of variants in noncoding regions. Unless supported by a strong clinically oriented diagnostic hypothesis, these variants remain largely under-analyzed due to the limited availability of *in silico* prediction tools for prioritization and the lack of functional assays for validation. We discuss here the current state of whole-genome analysis using the combined annotation-dependent depletion (CADD) score, one of the most efficient genome-wide prediction and most popular prioritization tool for genetic variants. In light of the worldwide participative ClinVar database that stores the disease classification of millions of human genetic variants, we evaluated the use of genomic region-specific thresholds to guide the geneticists in prioritizing noncoding region variants using the CADD score.

## Introduction

Major progress in genomics and bioinformatics led researchers and geneticists to determine the exact sequence of the human genome and capture its genetic diversity—ranging from thousands of structural variations (SVs) to millions of single-nucleotide variations (SNVs) and small insertions and deletions (indels) [1, 2]. However, accurately interpreting their consequences on the protein and the related biological processes remains a significant challenge [3] that requires a significant effort, especially when prioritizing variants for subsequent functional investigation [4], despite existing strong recommendations [5, 6]. Unlike coding variants, which are usually linked to protein function, variants in noncoding regions such as introns, untranslated regions (UTRs), and intergenic areas can still modulate gene regulation in complex and less predictable ways. This makes the prioritization of variants in noncoding regions particularly difficult, especially when there is no strong clinical suspicion or a first variant identified in the context of a recessive disease, which would make it possible to focus on a particular gene.

The combined annotation-dependent depletion (CADD) score emerged in 2014 [7] as an ensemble score integrating multiple annotation sources to predict the deleteriousness of human genetic variants whatever their category. Over the years, it has been refined with updated annotation sources and novel dedicated applications, improving the prediction of splice variants [8] and more recently regulatory variants [9]. Structural variants [10] were incorporated in the CADD-SV model, which was trained on a distinct dataset tailored for larger genomic alterations. The latest version (1.7) of CADD further enhances its predictive power by integrating evolutionary scale modeling for the assessment of protein-coding variants and by leveraging a convolutional neural network trained on human open chromatin sequences to improve the annotation of regulatory regions. The score is provided as a raw *C*-score and a Phred-like score scaled over ~9 million variants. While the latter offers lower resolution, it is more convenient and widely adopted by users. CADD assesses the principle of deleteriousness rather than pathogenicity; however, it is widely used to distinguish pathogenic from benign variants in human genetics. Although applying a single genome-wide CADD threshold is tempting, it has been discouraged by its developers since the initial publication [9]. Nevertheless, over time and maybe due to pressure from the human genetics community, indicative score ranges have been proposed (https://cadd.gs.washington.edu/info). For instance, a score above 20, which means that the variant belongs to the top 1% of the most deleterious predicted variants, was initially used. However, as it concerns an important part of the missense variants, a higher score, for instance 25, was preferred [11, 12]. If the use of this threshold appears pertinent for nonsynonymous variants (missense, frameshift, nonsense), it is not appropriate for the variants located outside of coding regions, for which the functional impact is more difficult to predict.

A typical whole-genome sequencing (WGS) leads to >4 million SNVs and 1 million indels, with ∼99% of noncoding variants, of which 8% are rare (allele frequency <1% according to gnomAD 3.0) [13]. In order to help the prioritization of these variants, we examined the distribution of the CADD Phred scores across the different noncoding regions to identify potential guidelines for optimizing their use in rare disease genetic analysis. A previous region-based approach, RAVA-FIRST, was developed to facilitate large-scale exploration of the noncoding genome. In this framework, the authors defined three broad genomic categories (“coding,” “regulatory,” and “intergenic”) and derived region-specific CADD score thresholds tailored to the expected functional impact within each category [14]. These region-specific thresholds have been used both for variant prioritization and in functionally informed burden tests, enabling a more context-aware analysis of genetic variations. A comparison between rare and common variants was also used to evaluate the clinical validity of the CADD score in a cancer panel for noncoding regions [15]. No difference was observed in the global score distribution, which does not mean that the CADD score is not discriminative, as we do not expect all rare variants to be deleterious. Thus, in our study, we preferred to use the ClinVar database [16] to assess the performance of the latest version of the CADD scores (v1.7) across various noncoding regions, complemented by real-life validation using an in-house dataset of *de novo* variants from patients with neurodevelopmental disorders.

## Materials and methods

### Implementation

All the functions needed for performing this analysis have been implemented in Python (v3.10.13) using several libraries for data analysis and visualization, as well as for statistical analysis, are listed in a Jupyter Notebook (https://jupyter.org) available at https://doi.org/10.5281/zenodo.17337880.

### Genomic regions

Genomic regions were defined based on the annotation from ANNOVAR (v20200608) [17]. The following nine categories have been considered: upstream region (1-kb region upstream of transcription start site) and downstream region (1-kb region downstream of transcription end site), 5′ and 3′ UTR, intronic and intergenic regions, as well as splicing (within 2 bp of a splicing junction), and exonic and intronic ncRNA.

### Variant annotation

WGS variant annotation was performed using HOWARD (Highly Open Workflow for Annotation & Ranking toward genomic variant Discovery), an in-house and open-source annotation tool developed by our team (Lamouche *et al.*, manuscript in preparation). HOWARD is implemented in Python and leverages the efficient Parquet file format (https://parquet.apache.org) for optimized data handling. HOWARD was set up for using snpEff (v.5.2a) and the corresponding database (v5.0) [18]. The software is freely available at https://github.com/bioinfo-chru-strasbourg/howard. A dedicated HOWARD configuration designed to facilitate streamlined integration of our results is available at https://github.com/bioinfo-chru-strasbourg/CADD_threshold/blob/master/CADDOnc.md.

### Databases and scores

#### CADD

The CADD dataset version 1.7 was downloaded directly from https://cadd.gs.washington.edu (GRCh38/hg38 genome build) [9]. A local installation of the CADD v1.7.2 scripts has been used for predicting variants not present in the database (https://github.com/kircherlab/CADD-scripts).

#### gnomAD

The gnomAD dataset v3.0 was downloaded from https://gnomad.broadinstitute.org/data (GRCh38/hg38 genome build) [13]. The common variant dataset used for a secondary analysis of benign variants was based on a randomly selected 10% of the data from the gnomAD v 4.1 database and had no overlap with the ClinVar pathogenic variants.

#### ClinVar

The ClinVar dataset release 20241120 was downloaded from https://ftp.ncbi.nlm.nih.gov/pub/clinvar/ in VCF format (GRCh38 genome build). All entries have been considered except mitochondrial variants, “unplaced” contigs, and conflicting variants. Pathogenic and likely pathogenic variants have been grouped together as well as benign and likely benign.

#### Comparison with the RAVA FIRST method

RAVA-FIRST annotations [14] were downloaded from https://lysine.univ-brest.fr/RAVA-FIRST. The dataset is composed of ∼8 billion SNVs and ∼45 million InDels with their ACS (adjusted CADD score). This dataset, only available in the GRCh37 version of the human genome, has been converted to GRCh38 using the LiftOverIntervalList and LiftOverVcf tools from GATK (v4.6.2) [19] with a success rate of 99.98% for SNVs and 99.78% for indels. Region data were processed the same way, and 99.74% of the entries were correctly converted to GRCh38. Following that, our WGS were annotated with HOWARD, and variants were filtered according to the RAVA-FIRST filtering strategy (ACS ≥ Median ACS per region). Not all genomic coordinates were covered, leading to ∼600 000 variants per genome without ACS.

## Results and discussion

### Using the ClinVar noncoding variant dataset

**Figure 1.**
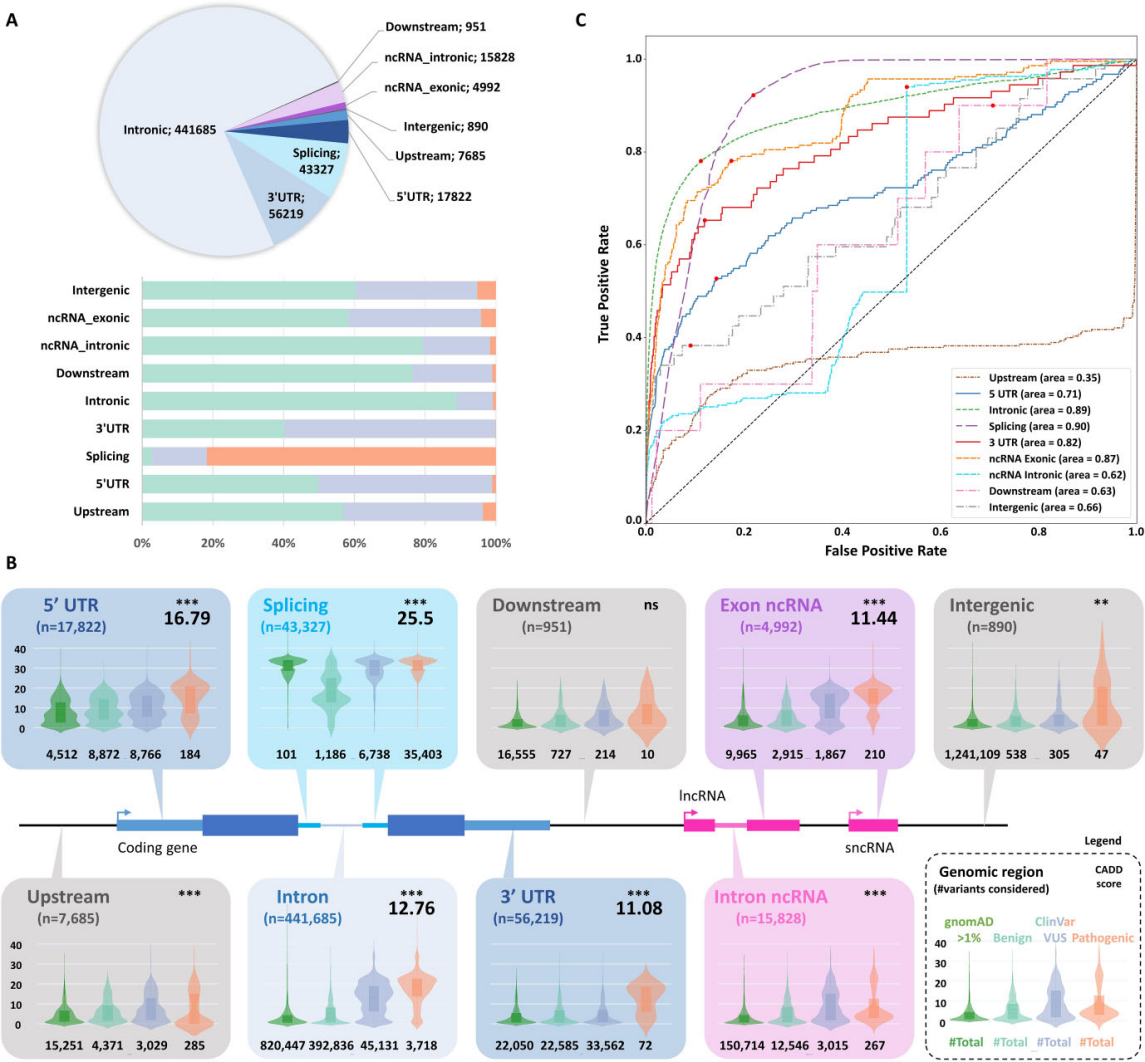
Distribution of the CADD score according to genomic regions and variant classification from the ClinVar database. (**A**) Pie chart representing the distribution of noncoding variants per region in ClinVar. Bar chart representing for each noncoding category the distribution of benign (defined as benign and likely benign variants; light green), VUS (light blue), and pathogenic variants (defined as pathogenic and likely pathogenic variants; light red). (**B**) CADD Phred score distribution by genomic regions and variant categories is shown as a combination of violin plots and boxplots. The total number of variants in each region is given below the region name (e.g. splicing: 43 327 variants). The number of variants in ClinVar for each category is given below the corresponding violin plot. Violin and boxplots are colored as follows: light green for ClinVar benign variants, light blue for VUS, light red for pathogenic, and dark green for the gnomAD frequent variants. Significant differences (Mann–Whitney *U* test, Benjamini–Hochberg FDR <0.05) between benign and pathogenic categories are highlighted like this: ns, non-significant; *< 0.05; **< 0.01; and ***< 0.001. If meaningful (i.e. AUC > 0.7) in panel (C), CADD thresholds are indicated in the top right corner. (**C**) ROC curves showing CADD score performance in distinguishing pathogenic from benign variants from ClinVar, with the optimal operating points identified using the ROC curve analysis marked by red circles.

ClinVar is one of the major publicly available sources of genetic variants with clinical interpretations. Submissions originate from a wide range of contributors, including diagnostic laboratories and research groups, which can occasionally lead to false positive classifications (either pathogenic or benign). However, this issue has been progressively mitigated over time through the implementation of expert review panels, community-based submissions, and consensus-driven reclassification efforts [20, 21]. As of the latest release, the ClinVar database consists of 2 876 049 variants with a clear clinical classification, distributed in 1 138 899 benign/likely benign (39.6%), 276 994 pathogenic/likely pathogenic (9.6%), and 1 460 156 variants of uncertain significance (VUS, 50.8%). Noncoding variants represent only 20.5% (589 399 variants) of all variants with a clinical classification, distributed as follows: 446 766 benign (39.2% of all benign), 40 196 pathogenic (14.5% of all pathogenic), and 102 627 VUS (7% of all VUS). To refine our analysis, we categorized these noncoding variants according to their genomic context: upstream and downstream, intergenic, 5′ and 3′ UTR, intronic, splicing, and ncRNA exonic and intronic regions (Fig. 1A). As expected, most of the noncoding variants are located in the intronic regions (*n* = 441 685; 78.6%). Next come variants in the 3′ UTR (*n* = 56 219; 10%), in splicing regions (*n* = 43 327; 7.7%), 5′ UTR (*n* = 17 822; 3.2%), ncRNA intronic (*n* = 15 828; 2.8%), upstream (*n* = 7685; 1.4%), and ncRNA exonic regions (*n* = 4992; 0.9%), while only a few variants are reported in intergenic (*n* = 890; 0.2%) and downstream regions (*n* = 951; 0.2%) (Fig. 1A and B).

### Distribution of CADD scores between genomic regions

Analysis of CADD score distributions per region revealed highly significant differences between benign and pathogenic variants for most genomic regions considered (Mann–Whitney *U* test, Benjamini–Hochberg FDR corrected *P*-value < 0.001), moderately significance for intergenic regions (adjusted *P*-value = .002), and no significant difference (adjusted *P*-value = 0.1554) for downstream regions, presumably because of the low number of pathogenic variants reported in this category (*n* = 5). In addition, the CADD score distribution for pathogenic variants differs significantly between the nine regions (*P*-values < 0.001 after correction), underlying the crucial need for region-specific thresholds when utilizing CADD scores for variant prioritization (Fig. 1B).

### Definition of region-specific CADD thresholds

Receiver operative characteristic (ROC) curves with Youden’s J statistic were employed to identify optimal thresholds balancing sensitivity (ability to identify true positives) and specificity (ability to identify true negatives) [22]. For categories with high variability in scores, precision–recall curves and F1 scores were used. Here, we prioritized a balance between precision (identifying true positives over false positives) and recall, with a weight of 0.4 given to Youden’s J statistic, emphasizing the importance of capturing true positives (pathogenic variants) [23]. The ROC curves (Fig. 1C) showed AUC (area under curve) values >0.8 for four regions (i.e. splicing, 3′ UTR, intronic, and ncRNA exonic). The 5′ UTR region is fairly useful with an AUC of 0.71, but the remaining regions fail with scores below 0.7. This analysis allowed us to define robust region-specific thresholds (Fig. 1B) for five out of the nine regions of interest (Fig. 1B). The highest score is given for splicing regions (CADD_Splicing_= 25.5), most probably due to major annotations and bioinformatics predictions available since version 1.6 of the CADD score. In contrast, other regions are not significant or with an AUC < 0.6 (e.g. CADD_Upstream_= 34, CADD_Downstream_= 0.82, CADD_Intergenic_= 11, CADD_ncRNAintronic_= 3.03). This likely reflects a lack of annotation sources for those regions, as well as the lack of reported variants in ClinVar. Given the relatively low values of these thresholds, they should be taken with caution. Interestingly, the distribution of CADD scores for VUS often falls between those of benign and pathogenic variants, which suggests that some VUS categories in ClinVar might harbor potentially pathogenic variants (Fig. 1A).

By incorporating ROC curve-derived thresholds tailored to different noncoding regions (Fig. 1B), we can more effectively flag potentially important variants. For example, splicing regions consistently require higher thresholds; meanwhile, regions such as UTRs and introns, while functionally significant, may be overlooked when applying overly stringent thresholds, leading to a potential loss of key regulatory variants. This analysis revealed significant variations across different regions, with CADD score thresholds ranging from 3 to 25 (Table 1).

**Table 1.** CADD score threshold according to genomic regions

Genomic region	CADD threshold	*P*-values	Significance	AUC
Upstream	34	5.5983e−18	***	0.347
5′ UTR	16.79	5.2879e−23	***	0.714
Intronic	12.76	0.0	***	0.886
Splicing	25.5	0.0	***	0.904
3′ UTR	11.08	1.2086e−20	***	0.820
ncRNA exonic	11.44	4.3687e−72	***	0.872
ncRNA intronic	3.03	1.9291e−11	***	0.620
Downstream	0.82	1.6134e−01	n.s.	0.629
Intergenic	11.0	2.2073e−04	**	0.664

CADD thresholds indicated per genomic regions. Significance and usability (AUC > 0.7) are indicated in the respective columns. Significance between benign and pathogenic categories is assessed using a Mann–Whitney *U* test and Benjamini–Hochberg FDR < 0.05. n.s., non-significant; **<0.01; ***<0.001; AUC, area under the curve.

### Validation using common variants

Despite the application of stringent criteria for the ClinVar variants (e.g. exclusion of conflicting classifications), some benign variants may still be misclassified, often due to overreliance on allele frequency or computational prediction tools. Among the 446 766 benign noncoding variants in ClinVar, only 151 274 (33.9%) are frequent variants (allele frequency >1%) according to gnomAD (Supplementary Table S1). To improve the robustness of our results, we replaced the ClinVar-derived benign variants with a set of randomly selected common variants from gnomAD v4.1 (AF > 1%) and repeated the computations using this alternative dataset. This resulted in a total of 2 280 704 variants, covering all noncoding categories with a predominance of intronic and intergenic variants (Supplementary Fig. S1A). Interestingly, the overall distribution of CADD scores across genomic regions remained largely consistent with that observed using the ClinVar dataset (Fig. 1B), supporting the robustness of our observations. An exception was noted in the “splicing” category, although it involved a very limited number of variants (*n* = 101). The distribution had high CADD scores that may include potentially hypomorphic alleles (only 21 are OMIM genes of which 14 are of recessive inheritance) or variants in non-disease genes (*n* = 80). The observed region-specific thresholds were only slightly modified (Supplementary Table S2). Since ClinVar remains the more curated and clinically interpretable dataset, we retained the thresholds derived from ClinVar for subsequent analyses.

### Confirming the efficiency of using region-specific CADD thresholds on real WGS cases

As an example, the recent implication of small nuclear RNA as a major cause of neurodevelopmental (*RNU4-2*) [24] or neurosensory (*RNU4-2, RNU6*) [25] disorders exemplifies the importance of a proper integration of the noncoding genome annotation and prioritization. We took advantage of trio WGS of two patients with neurodevelopmental disorder (NDD) caused by *RNU4-2* variants to test the usefulness of the proposed region-specific CADD thresholds. We identified on average ∼5.8 × 10^6^ variants in the noncoding regions per genome (>99% of the variants), of which ∼455 000 are rare and ∼35 200 have a higher CADD score value than their region-specific thresholds (Supplementary Table S3). Among them, a reasonable number of variants were found in the 5′ (*n* = 42 on average) and 3′ (*n* = 172) UTRs, in splicing regions (*n* = 8), and in ncRNA exonic (*n* = 132), while an important number of variants were still identified in downstream (1484), intronic (1593), ncRNA intronic (7566), and intergenic (24 293) regions (Supplementary Table S3). As >70% of monogenic forms of NDD are known to be caused by *de novo* variants, this criterion is usually used when analyzing pangenomic data from these patients. Then, filtering for *de novo* variants (*n* = 80/WGS) revealed only ∼8 variants having a CADD score above their region-specific threshold out of which one was the recurrent *RNU4-2* variant (n.64_65insT, CADD score = 20.8, which is largely above the CADD_ncRNAexonic_ threshold of 11.44) [26].

To evaluate our updated method, we compared it to the RAVA-FIRST approach. While both strategies rely on regional annotation, RAVA-FIRST applies region-based adjusted scores, whereas our method introduces variant-level thresholds specific to each genomic category. As described above, our thresholds allowed us to retain ∼35 200 rare noncoding variants, compared to ∼94 700 variants identified using RAVA-FIRST (Supplementary Table S2). Notably, the pathogenic *RNU4-2* variant could not be recovered initially by RAVA-FIRST. The available precomputed RAVA-FIRST dataset does not include novel indels that would require recomputation. Several factors may explain this discrepancy, including differences in genome assembly versions (GRCh37 versus GRCh38), the use of different CADD versions (1.4 in RAVA_FIRST versus 1.7 in our analysis), and the lack of available tools to recompute adjusted CADD scores in the RAVA-FIRST framework for newly observed variants.

Our approach tailors CADD score application to the unique features of noncoding regions, facilitating the identification of novel pathogenic variants in whole-genome analysis by geneticists. Our results revealed significant variability in CADD score distributions across different noncoding regions, making it clear that region-specific thresholds would be useful for human geneticists for accurate prioritization. These thresholds can serve as conservative cutoffs, below which the likelihood of pathogenicity is low and variants should be deprioritized or interpreted with increased caution. By demonstrating the importance of using region-specific thresholds for CADD scores, we improve noncoding variant classification and prioritization. Although useful, some categories lack a sufficient number of variant for definitive thresholds. These thresholds will be refined over time as novel interesting variants will be reported in ClinVar, further enhancing the accuracy of noncoding variant classification.

## Supplementary Material

lqaf157_Supplemental_Files

## Data Availability

Data generated or analyzed during this study are included in the published article. The source code and data are available from https://doi.org/10.5281/zenodo.17337880.
