# Supplementary material for: Genome region aware CADD thresholds for noncoding variant prioritization
Source: NAR Genom Bioinform. 2025 Nov 19;7(4):lqaf157. doi: 10.1093/nargab/lqaf157 (PMC12629842; doi:10.1093/nargab/lqaf157)
Supplement: lqaf157_Supplemental_Files [file lqaf157_supplemental_files.docx]

**Definition of region specific CADD thresholds using common variants from gnomAD**

The gnomAD dataset is composed of randomly selected 10% of the v4.1 dataset leading to a total of 2,280,704 frequent and non-pathogenic variants from ClinVar (Supplementary Figure 1A). The genomic distribution represent a dramatic increase in the “Intergenic” and “Intronic” regions. Overall the distribution of those variants are very similar to those of the ClinVar dataset (Figure 1B) and the thresholds and AUC have been recomputed (Supplementary Table 2 and supplementary Figure 1B).

The “Downstream” region remains non-significant while the other regions remains at the same level of significance in their ability to discriminate the pathogenic from the benign variants (here AF>1%). ROC curves allowed to determine novel AUC for each regions. The most dramatic shift concern the intronic region where the computed threshold decrease from 12.76 to 0, correlated with the best AUC (0.923). However, this drastic change could be explained by the highly imbalanced dataset (3,718 pathogenic vs 820,447 frequent variants). This extreme imbalance forces the optimization algorithm to minimize false positives among 824,000 frequency-based "benign" variants, mathematically converging toward a threshold (0.001) that accepts virtually all variants and provides no clinical filtering capacity. The misleading high AUC (0.923) primarily reflects correct classification of the overwhelming majority class rather than true discriminatory power, demonstrating how population frequency assumptions create statistically optimal but clinically meaningless results.


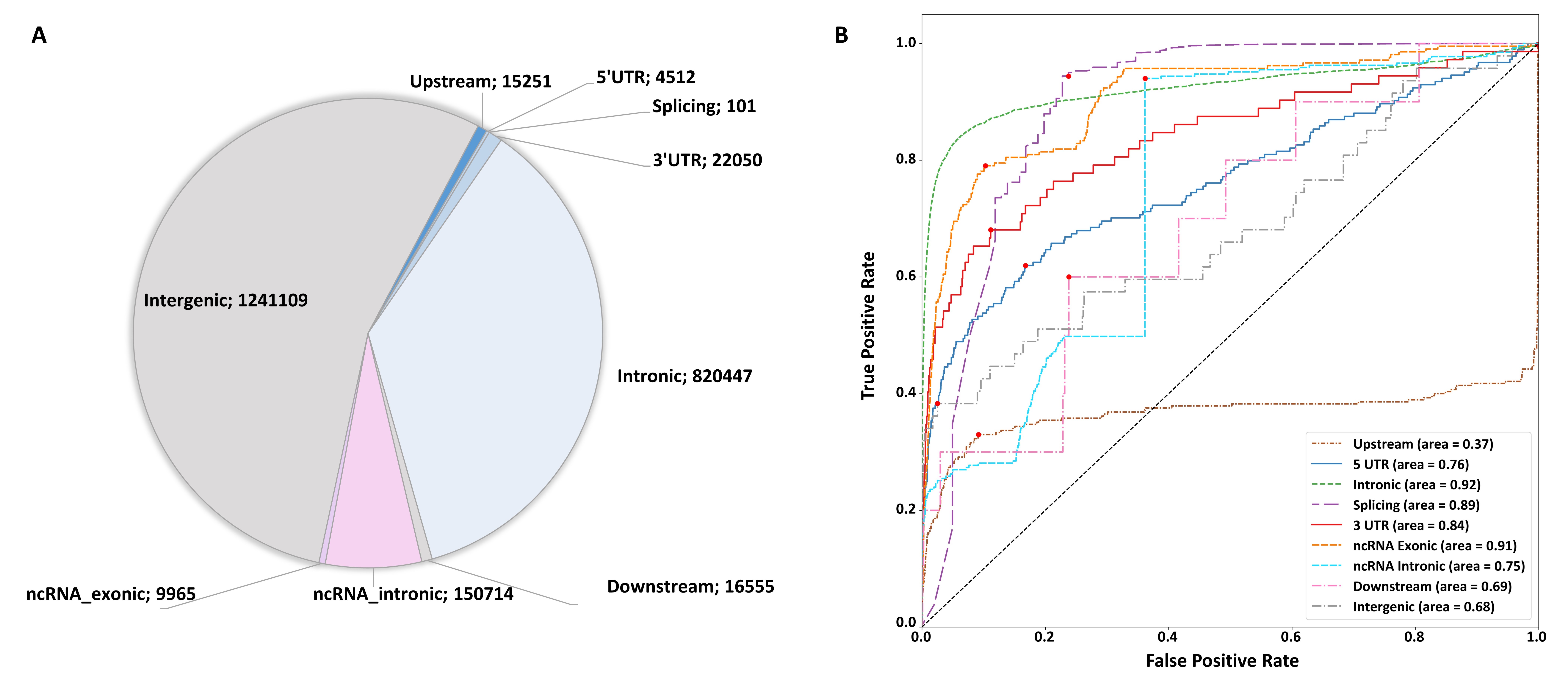


**Supplementary Figure 1.** **A**. Distribution of a randomly selection of variants (10%) in gnomAD (v4.1) with an AF>1% per genome categories. **B**. Receiver operating characteristics (ROCs) curves showing CADD score performance in distinguishing pathogenic (ClinVar) from frequent variants (AF>1%) from gnomAD, with the optimal operating points identified using the ROC curve analysis marked by red circles.

|  | **No AF** | **AF > 1%** | **AF <= 1%** | **Total** |
| --- | --- | --- | --- | --- |
| **Upstream** | 256 | 3677 | 462 | 4395 |
| **5'UTR** | 1560 | 4292 | 3005 | 8857 |
| **Splicing** | 330 | 237 | 464 | 1031 |
| **3'UTR** | 1285 | 15955 | 5375 | 22615 |
| **Intronic** | 150716 | 121171 | 121377 | 393264 |
| **Downstream** | 21 | 484 | 73 | 578 |
| **ncRNA_intronic** | 4638 | 4183 | 3745 | 12566 |
| **ncRNA_exonic** | 695 | 1022 | 1199 | 2916 |
| **Intergenic** | 51 | 253 | 240 | 544 |
| **Total** | 159552 | 151274 | 135940 | 446766 |
|  | 35.6% | 33.9% | 30.4% |  |

**Supplementary Table 1.** Distribution of the allele frequencies of the ClinVar benign variants according gnomAD (v3). Rare variants are defined by an allele frequency <1%.

| **Genomic region** | **CADD threshold** | **p-values** | **Significance** | **AUC** |
| --- | --- | --- | --- | --- |
| **Upstream** | 10.85 | 3.9532e-14 | *** | 0.369 |
| **5' UTR** | 14.35 | 2.1701e-33 | *** | 0.762 |
| **Intronic** | 0.0 | 0.0 | *** | 0.923 |
| **Splicing** | 24.8 | 8.0161e-43 | *** | 0.894 |
| **3' UTR** | 9.33 | 3.7361e-23 | *** | 0.839 |
| **ncRNA Exonic** | 10.85 | 9.1473e-90 | *** | 0.906 |
| **ncRNA Intronic** | 3.03 | 5.1013e-46 | *** | 0.753 |
| **Downstream** | 4.43 | 3.3008e-02 | n.s. | 0.695 |
| **Intergenic** | 12.31 | 1.3978e-05 | ** | 0.684 |

**Supplementary Table 2. CADD score thresholds according to genomic regions using the gnomAD and ClinVar datasets.** CADD thresholds indicated per genomic regions based on the pathogenic variants from ClinVar and the common variants from gnomAD (AF≥1%). Significance and usability (AUC>0.7) are indicated in the respective columns. Significance between benign and pathogenic categories is assessed using a Mann-Whitney U test, Benjamini-Hochberg FDR < 0.05. ns non-significant, *<0.05, **<0.01, ***<0.001. AUC: Area Under the Curve

|  | **WGS_1** | | | | |  | **WGS_2** | | | | |
| --- | --- | --- | --- | --- | --- | --- | --- | --- | --- | --- | --- |
|  | **#All** | **#Rare** | **#Rare CADD** | **#Rare Denovo CADD** | **#Rare RAVA** |  | **#All** | **#Rare** | **#Rare CADD** | **#Rare Denovo CADD** | **#Rare RAVA** |
| **Upstream** | 38120 | 2517 | 0 | 0 | 1103 |  | 37494 | 2283 | 0 | 0 | 990 |
| **5'UTR** | 8909 | 452 | 47 | 0 | 184 |  | 8632 | 350 | 36 | 0 | 146 |
| **Splicing** | 206 | 25 | 7 | 0 | 12 |  | 195 | 23 | 9 | 0 | 10 |
| **3'UTR** | 51922 | 2221 | 187 | 0 | 881 |  | 50606 | 1898 | 157 | 0 | 725 |
| **Intronic** | 2022243 | 99608 | 1793 | 2 | 36998 |  | 1996965 | 89220 | 1392 | 2 | 32825 |
| **Downstream** | 39798 | 2124 | 1538 | 0 | 751 |  | 38931 | 1957 | 1429 | 1 | 703 |
| **ncRNA_intronic** | 378475 | 20755 | 7874 | 2 | 7093 |  | 370582 | 18656 | 7257 | 2 | 6339 |
| **ncRNA_exonic** | 21922 | 1257 | 142 | 1 | 471 |  | 21282 | 1122 | 122 | 1 | 480 |
| **Intergenic** | 3289661 | 330015 | 24236 | 2 | 52509 |  | 3238977 | 333148 | 24350 | 2 | 47354 |
| **Exonic** | 28039 | 1734 |  |  |  |  | 27464 | 1567 |  |  |  |
| **Others** | 1789 | 186 |  |  |  |  | 1743 | 156 |  |  |  |
| **Total non-coding** | 5851256 | 458974 | 35824 | 7 | 100002 |  | 5763664 | 448657 | 34752 | 8 | 89572 |
| **Total** | **5882873** | **461080** |  |  |  |  | **5794614** | **450536** |  |  |  |

**Supplementary Table 3.** Detailed number of variants identified by genomic regions for the 2 genomes of interest (numbered 1 and 2). Rare variants are defined by an allele frequency <1% (gnomAD v3). RAVA corresponds to the annotated variants using the RAVA-First method.
